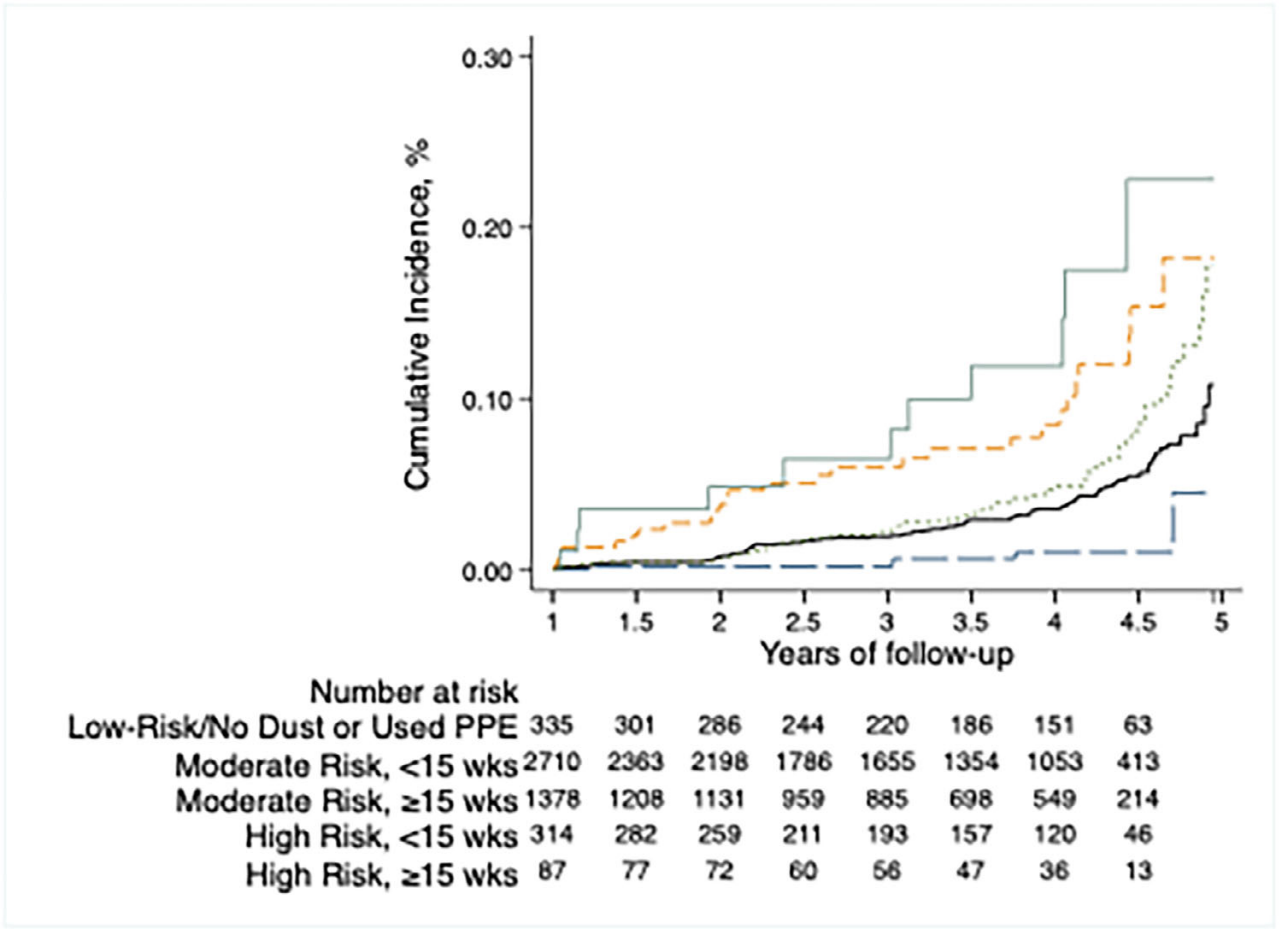# Incidence of dementia before age 65 years following World Trade Center exposure: A prospective study of cognition in general responders

**DOI:** 10.1002/alz.089161

**Published:** 2025-01-09

**Authors:** Sean A.P. Clouston, World Trade Center Aging Research Team

**Affiliations:** ^1^ Stony Brook University, Stony Brook, NY USA

## Abstract

**Background:**

The goal of this study was to determine the incidence of dementia before age 65 years in a prospective study of WTC responders, and compare incidence among responders with severe exposures to debris versus responders not exposed to building debris or those who wore personalized protective equipment (PPE).

**Methods:**

Data were collected in a congressionally mandated academic occupation‐based medical monitoring program available to all verified WTC‐exposed responders residing on Long Island, NY, most of whom are currently <65 years of age. WTC responders aged ≤60 years without dementia at the time of their first cognitive assessment were followed every 18 months on average, for up to five years. We determined exposure severity based on responses to a detailed questionnaire of WTC‐related exposures and exposure‐related work activities that proxied exposures to fine particulate dust and potentially neurotoxic chemicals/dust/debris, duration of work, and the use PPE. We categorized exposure levels using an ordinal variable with five categories ranging from low to severe exposure. The incidence of dementia before age 65 years was the outcome, and was diagnosed following standard guidelines.

**Results:**

Of 9,891 responders in the cohort, 5,010 were eligible for inclusion in this study of cognitive function (median age 53; 437 (8.72%) female), and there were 228 cases of dementia identified during 15,913.1 person‐years of follow‐up. Increasing severity and duration of exposure reported at the WTC sites revealed incremental increases in the incidence of dementia (Figure 1). After adjusting for social, demographic, and relevant medical factors, each unit increase in exposure severity was associated with increased incidence of dementia (risk difference = 39.42 [11.12‐139.70] P<0.001; hazards ratio = 1.42 [1.18–1.71], P<0.001).

**Conclusions:**

Among WTC responders who survived to participate in a longitudinal follow‐up study of cognition from 2014‐22, a higher level of reported exposure to dust/debris, when compared with the lowest reported exposure levels or use of PPE, was significantly associated with a higher risk of dementia before age 65 years.